# Generating statistics from health facility data: the state of routine health information systems in Eastern and Southern Africa

**DOI:** 10.1136/bmjgh-2019-001849

**Published:** 2019-09-29

**Authors:** Abdoulaye Maïga, Safia S Jiwani, Martin Kavao Mutua, Tyler Andrew Porth, Chelsea Maria Taylor, Gershim Asiki, Dessalegn Y Melesse, Candy Day, Kathleen L Strong, Cheikh Mbacké Faye, Kavitha Viswanathan, Kathryn Patricia O’Neill, Agbessi Amouzou, Bob S Pond, Ties Boerma

**Affiliations:** 1 International Health, Johns Hopkins University Bloomberg School of Public Health, Baltimore, Maryland, USA; 2 Department of Research, African Population and Health Research Center, Nairobi, Kenya; 3 Division of Data, Research and Policy, Data and Analytics Section, UNICEF, New York City, New York, USA; 4 Data Analytics and Delivery, World Health Organization, Geneva, Switzerland; 5 Department of Community Health Sciences, University of Manitoba, Winnipeg, Manitoba, Canada; 6 Health System Trust, Westville, South Africa; 7 Maternal, Newborn, Child and Adolescent Health Department, World Health Organization, Geneva, Switzerland; 8 West Africa Regional Office, African Population and Health Research Center, Nairobi, Kenya; 9 Information Evidence and Research, World Health Organization, Geneva, Switzerland; 10 Independent Consultant, Portland, Oregon, USA; 11 Centre for Global Public Health, University of Manitoba, Winnipeg, Manitoba, Canada

**Keywords:** routine health information systems, data quality assessment, DHIS2, Eastern and Southern Africa

## Abstract

Health facility data are a critical source of local and continuous health statistics. Countries have introduced web-based information systems that facilitate data management, analysis, use and visualisation of health facility data. Working with teams of Ministry of Health and country public health institutions analysts from 14 countries in Eastern and Southern Africa, we explored data quality using national-level and subnational-level (mostly district) data for the period 2013–2017. The focus was on endline analysis where reported health facility and other data are compiled, assessed and adjusted for data quality, primarily to inform planning and assessments of progress and performance. The analyses showed that although completeness of reporting was generally high, there were persistent data quality issues that were common across the 14 countries, especially at the subnational level. These included the presence of extreme outliers, lack of consistency of the reported data over time and between indicators (such as vaccination and antenatal care), and challenges related to projected target populations, which are used as denominators in the computation of coverage statistics. Continuous efforts to improve recording and reporting of events by health facilities, systematic examination and reporting of data quality issues, feedback and communication mechanisms between programme managers, care providers and data officers, and transparent corrections and adjustments will be critical to improve the quality of health statistics generated from health facility data.

**Summary box**

- Routine health information systems are a potential source of data to generate health statistics and indicators to track national and subnational progress towards universal health coverage and to inform planning and assessments of progress and performance.
- The introduction of web-based digital platforms (DHIS2) was a notable development leading to better standardisation of data collection and to gradual improvements in data quality, but there are persistent data quality issues.
- Using population projections from National Statistical Offices for target populations often leads to improbable coverage statistics, but several countries are exploring alternative methods.
- Endline analysis is an important component of continuous efforts to improve facility-based statistics, including systematic ways to examine and present data quality issues and use of transparent adjustment procedures.
- National analysts in the Ministry of Health, public health institutions and national statistical offices need to have access to an optimal set of tools and skills to analyse and synthesise health facility data and produce the best possible statistics with well-documented audit trails.

## Introduction

Routine health information systems (RHIS), based on data reported by health facilities, are an important source of health statistics that feature prominently in national and subnational health plans and programme.^1–3^ Multiple indicators generated by the RHIS data can be used to track national and subnational progress towards universal health coverage, often in combination with household survey and other data. Scorecards and dashboards are increasingly popular tools to visualise the statistics based on health facility data, aiming to facilitate the interpretation, communication and use of data.^4^

Countries and development partners have been investing in the improvement of the data generation and use through the RHIS.^4 5^ A notable development is the introduction of the District Health Information System (DHIS), which is an open-source software platform for reporting, quality checks, visualisation, analysis and dissemination of data for all health programme.^6^ From 2010 onwards, an increasing number of countries began to introduce the web-based DHIS2 platform, and today many countries are using this electronic platform.^7^

Common RHIS data-based indicators include causes of death and morbidity patterns among persons using health services, health service utilisation and efficiency indicators, as well as a range of program-specific indicators on the coverage of interventions.^8^ Several programmes such as immunisation and HIV have been relying extensively on facility data-based coverage statistics for country and global monitoring.^9–11^

Studies have shown multiple issues regarding the quality of data generated by health facilities that affect the credibility and utility of RHIS-based statistics at local and national levels.^12–18^ The main challenges are associated with incomplete and inaccurate reporting of events, as well as problems with defining accurate denominators (ie, target populations) for the computation of coverage statistics.

This paper describes the situation in 14 countries in Eastern and Southern Africa in 2017, based on an analysis project involving teams of Ministry of Health and country public health institutions analysts, organised by the African Population Health Research Centre, Countdown to 2030 for Women’s, Children’s and Adolescents’ Health, WHO and UNICEF. The focus was on ‘endline’ analysis where all relevant health facility data are compiled and systematically assessed, including assessment and adjustment for incomplete reporting, detection and correction of extreme outliers, assessment and revision of denominators, comparison with survey-based results and computation of statistics based on the adjusted data set. These analyses were done in MS Excel 2013, using data exported from the DHIS2 country databases.

## Country contexts

The 14 countries produced data for 937 subnational units (primarily districts) with an average population size of 274 278 per unit, ranging from less than 100 000 in districts in Eritrea, Botswana and Namibia, to over one million in Kenya’s counties and South Sudan’s states (table 1). All countries were using the RHIS data for statistical reporting. In 2017, the RHIS data were underpinning annual health statistical reports (10 countries), health system performance assessment reports (7), national health statistical profiles (8) and district health statistical profiles (10). Mozambique and Zambia produced all four outputs.

**Table 1:** General characteristics and reporting completeness, national (%) and subnational units, 2017

Country	Population (2017)	Type of administrative unit	Number of subnational units	Average population per unit	Number of health facilities	Reporting rate	Per cent of subnational units with ≥90% reporting rates
Botswana	2 218 739	District	27	82 176	1702	69	22
Burundi	9 978 120	District	46	216 916	1253	97	90
Eritrea	3 781 759	District	58	65 203	398	96	92
Kenya	48 576 374	County	47	1 033 540	10 753	82	32
Lesotho	1 941 941	District	10	194 194	290	76	45
Malawi	17 373 185	District	29	599 075	719	86	66
Mozambique	26 863 901	District	161	166 857	1886	94	74
Namibia	2 348 872	District	35	67 111	407	71	41
Rwanda	11 809 295	District	30	393 643	818	96	88
South Sudan	11 837 437	State	10	1 183 744	1597	49	0
Tanzania*	52 619 314	Council	184	285 975	7403	99	98
Uganda	37 741 300	District	128	294 854	7056	99	95
Zambia	16 180 840	District	109	148 448	2996	96	88
Zimbabwe	13 727 493	District	63	217 897	1778	99	100
Total/median**	256 998 570		937	**274 278**	**1702**	**95**	**81**

* 2018, reference year for Tanzania.

** Values in bold are median values

Routine service data are collected on paper by most health facilities and reported to the district on a monthly basis. The paper-based facility reports are entered into a computer in the districts and are accessible at the district and national levels. Among the 14 countries, 13 use DHIS2 for most data and programmes, while South Sudan uses DHIS V.1. In 8 of the 14 countries, DHIS has been operational for at least 5 years.

## Completeness of reporting

The reporting rates are based on the number of reports received divided by the expected number of reports from all listed facilities in the RHIS (master facility list), including public, non-government organisation (NGO) and private-for-profit facilities. Variation in reporting rates between districts or over time will affect performance and trend analysis of coverage and other indicators. Most countries ignore reporting rates in the analysis of differences or trends in indicators, which means that it is assumed that non-reporting facilities are not providing any services. If reporting completeness is well over 90%, the impact of this assumption is limited. Some country analyses, however, adjusted for incomplete reporting, using assumptions about level of activity in non-reporting facilities compared with those that reported.^19^ These adjustments to the data set need to be made in a transparent manner, creating an adjusted data set without modifying the underlying reported data.

Reporting rates have improved to high levels in most countries, which was corroborated by other studies (table 1).^20 21^ A few countries use a harmonised monthly reporting form that includes all health services, but most rely on a separate set of reporting forms for each service. In case of multiple forms, we computed the average of the reporting rate for outpatient department (OPD) services, antenatal care; institutional delivery and immunisation services. Very low reporting rates were observed in South Sudan (49%), often related to armed conflict, but the overall picture shows high reporting rates with eight countries exceeding 90%.

## Accuracy of reported health facility data

The accuracy of the data (the extent to which the data reflect the true numbers) can be assessed through endline analyses and facility assessments with data verification. The latter method relies on facility visits and record reviews to compare reported data with source documents within the facility and is discussed elsewhere.^10^ DHIS2 has now incorporated a WHO data quality module that can be used to identify outliers and assess internal and external consistency.^22^ By 2018, 6 of the 14 countries were using this tool within DHIS2.

The internal consistency of the health facility data is examined with three data quality metrics: presence of major outliers, variation for selected indicators over time and consistency between interventions. Major outliers for monthly aggregated data should be detected and corrected at the early stages of facility and district reporting. At the endline analysis stage, a final check for any extreme outliers is important as the impact on the results can be very large. Errors should be corrected with a clear audit trail (ie, a record of what has been changed). To confirm whether extreme outliers are in fact errors, external factors will need to be considered such as prolonged stock-outs (eg, vaccines), the seasonality of diseases (eg, malaria) or population migration (eg, conflicts, refugees). In the country data sets for the most recent year of the 14 countries, extreme outliers were identified using a modified Z-score, using 3.5 SD from the median based on the previous 3 years as threshold.^22 23^ In general, extreme outliers were rare (country median 6%), but cannot be ignored (table 2).

**Table 2:** Health facility data quality of reported event data, 2017: extreme outliers, consistency over time and internal consistency between interventions

Country	Extreme outliers for ANC, DPT and OPD			Consistency over time*	Internal consistency between interventions†		
	% of national values that are outliers‡	% of units with no outliers (last 12 months)‡	% of units with no outliers (last 3 years)§	% of units with consistent time trends	ANC1–DPT1: % difference from expected ratio	DPT1–DPT3: % difference from expected ratio	% of units with good consistency for both indicator pairs
	(a)	(b)	(c)	(d)	(e)	(f)	(g)
Botswana	6	57	75	43	–	–	–
Burundi	5	59	50	43	17	2	47
Eritrea	6	52	65	40	28	7	16
Kenya	3	81	42	37	1	1	57
Lesotho	6	57	34	47	5	14	25
Malawi	10	62	60	40	76	6	35
Mozambique	7	48	–	–	179	11	2
Namibia	8	46	62	23	7	4	37
Rwanda	7	51	77	40	7	0	63
South Sudan	7	53	80	33	67	57	–
Tanzania	6	54	47	43	5	1	38
Uganda	6	58	–	–	8	12	18
Zambia	7	52	37	43	3	6	40
Zimbabwe	5	60	63	37	5	3	5
Median	6	55	61	40	7	6	36
IQR	1	6	22	7	22	9	24

(a) Average percentage of outliers for ANC1, DPT3 and OPD; (b) average percentage for ANC1, DPT3 and OPD; (c) average percentage for ANC1, DPT1 and OPD.

*Good consistency over time defined as modified z-score lower than 1.

†Percentage difference between routinely reported ratio and survey: values were classified as good (<5), different (5–15) or very different (>15).

‡Outliers defined as modified z-score greater than 3.5; units are second-level administrative divisions in each country (district, county, etc).

§Outliers defined as modified z-score greater than 2; units are administrative divisions in each country (district, county, etc).

ANC, antenatal care; DPT, diphtheria-pertussis-tetanus; OPD, outpatient department.

There is usually only limited year-to-year variation in the reported numbers of interventions for, for example, first antenatal care visit (ANC1), first dose of diphtheria-pertussis-tetanus vaccine (DPT1) and OPD visits. We expect a modest annual increase in the number of people receiving services due to population growth (about 1.4% per year in Southern Africa and 2.8% per year in Eastern Africa) and potential improvements in service coverage.^24^ To assess year-to-year variation, we used the modified Z-score with 2 SD from the median for the three preceding years to identify potential inconsistencies. There was considerable variation for the national and district levels in several countries (table 2). The median percentage of districts with no outliers was 61% (IQR: 22%).

Internal consistency of interventions was assessed between ANC1 and DPT1 vaccination (recommended at 6 weeks of age) and between the first and the third doses of DPT vaccine. The metric is computed as the absolute difference in the ratio of expected numbers of ANC1 and DPT1 from the ratio of reported numbers of ANC1 and DPT1. The expected ratio is obtained from the population coverage rates in a recent household survey such as Demographic and Health Survey or Multiple Indicator Cluster Survey. Good internal consistency is defined as a small difference (≤5%) between reported numbers of ANC1 and pentavalent1/DPT1. The accuracy of reported numbers of DPT1 and DPT3 was assessed similarly. Table 2 presents the results of the assessment, showing substantial quality issues for almost all countries, especially for consistency between ANC1 and DPT1. Mozambique presents an extreme outlier (179%), which is due to major over-reporting of ANC1, as the expected number of births is closer to the DPT1 vaccinations. That must be due to a systematic error in the system. Online supplementary annex 1 shows the ratio of the reported number of ANC1 by the reported number of DPT1 over time and by country.

10.1136/bmjgh-2019-001849.supp1 Supplementary data

## Target populations

The national population census provides data on the population by age and sex, which are projected using assumptions about fertility, mortality and migration. The longer ago the census, the less accurate the projections. In 2018, the median year of the most recent census used for the population projections in the 14 countries was 2009 (table 3). Only Uganda had projections based on a census conducted less than 5 years ago. Two countries had conducted censuses from 2016 to 2017 (Lesotho, Mozambique), but population projections were not yet available by November 2018.

**Table 3:** Most recent census and coverage rates of ANC1, BCG and DPT1 in most recent household surveys (%)

Country	Year of last census	Survey	ANC1*	BCG*	DPT1*
Botswana	2011	MICS-2000	92.5	97.9	95.6
Burundi	2008	DHS-2016	99.3	97.7	99.2
Eritrea	None	DHS-2002	71.6	91.4	90.6
Eswatini	2017†	MICS-2014	98.7	98.4	96.4
Kenya	2009	DHS-2014	95.3	96.7	97.5
Lesotho	2016†	DHS-2014	95.0	98.0	98.3
Malawi	2008	DHS-2015	94.9	97.6	97.4
Mozambique	2017†	DHS-2011	90.7	91.1	91.3
Namibia	2001	DHS-2013	96.6	94.2	92.7
Rwanda	2012	DHS-2015	99.1	98.9	99.1
South Africa	1996	DHS-2016	93.9	92.5	91.2
South Sudan	2008	MICS-2010	42.8	34.4	28.1
Tanzania	2012	DHS-2015	97.9	96.0	97.0
Uganda	2014	DHS-2016	97.5	96.3	94.9
Zambia	2010	DHS-2013	95.4	94.9	95.9
Zimbabwe	2012	DHS-2015	92.0	89.9	89.5
Median	2009		95.3	96.0	95.9

*Coverage statistics from last survey.

†Projection data not yet available by mid-2018.

ANC, antenatal care; BCG, Bacille de Calmette and Guerin; DHS, Demographic and Health Survey; DPT, diphtheria-pertussis-tetanus; MICS, Multiple Indicator Cluster Survey.

Population projections were provided by National Statistical Offices. Based on our assessment of the population growth rates and parameters used to compute the target populations, a constant population growth rate for all years was used in half of the 14 countries (Burundi, Eritrea, Malawi, Rwanda, South Sudan, Tanzania and Zimbabwe). The crude birth rate (CBR) is a critical input for the RHIS, but very few countries used results on birth rates from recent national surveys, and none used subnational birth rates to estimate target populations.

The population projections from National Statistical Offices are the standard tool for obtaining target populations, but additional methods are needed to supplement those estimates for health statistical analyses. Censuses may have inaccuracies (such as an undercount of some areas) and projections can deviate substantially from reality, especially if there is substantial migration.

Frequent changes to administrative boundaries (increasing the number of districts and provinces/regions) were common, further complicating population projections. In addition, census-based projections can be a challenge since people may seek care from health facilities outside their district of residence. This has also been referred to as numerator/denominator mismatch.^9 12 15 25^ The result can be that some districts have coverage that is significantly greater than 100% while other districts and health facilities have very low coverage when census projections are used to estimate denominators.

To explore the consistency of denominators, we compared the results from four methods to estimate the number of live births at the national level: the number of births projected by the National Bureau of Statistics, the number of births computed from the total population projections by the National Bureau of Statistics and the CBR from the most recent household survey, the number of births derived from the reported number of DPT1 vaccinations reported and from the reported number of first antenatal visit through the RHIS, both adjusted for incomplete reporting and for non-use of services. The latter two methods use the facility data for high-coverage interventions such as ANC1 visit, Bacille de Calmette and Guerin (BCG) or DPT1 vaccination to obtain estimates of the target population size.^19^ The accuracy of these alternative denominators depends primarily on the quality of reporting by the health facilities. In addition to the data quality assessments presented in this paper, external validation of coverage estimates obtained with facility data-based denominators with survey-based statistics, for instance third dose of DPT1 vaccine, four antenatal visits or institutional delivery, provides critical information on the quality of reporting in the RHIS. Data quality and primarily over-reporting of events such as vaccinations are particular concern, in some cases, if there are incentives for vaccinating children.^26^ Studies in Kenya and Tanzania are examples of the use of facility data-derived denominators for coverage estimates.^19 27^

**Figure 1:**
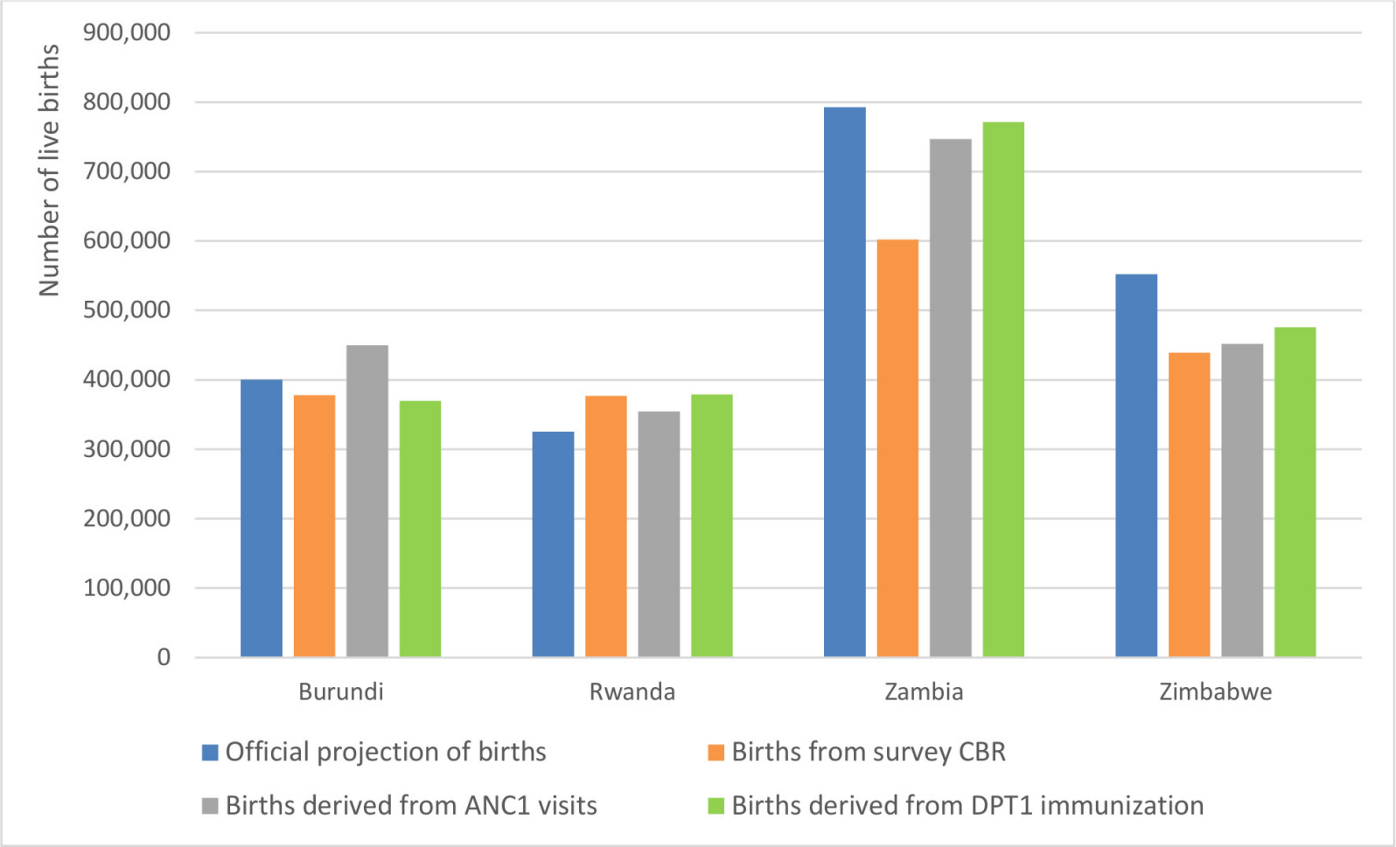
Estimated number of live births (denominators) for coverage statistics, projections and facility data, selected countries, national level, 2017. ANC, antenatal care; CBR, crude birth rate; DPT, diphtheria-pertussis-tetanus.

Figure 1 shows substantial differences between the methods of estimating live births at the national level in selected countries, illustrating the challenge of obtaining accurate denominators for facility data-based analysis. This challenge is magnified if we consider district-level denominators. The projections, whether official projections or estimates obtained from recent CBR data, provide denominators that lead to problematic results. Overall, one-third of districts (median 33%, IQR=48%) and nearly half of countries (median 46%, IQR=25%) had DPT1 coverage rates exceeding 100% based on the birth projections and the CBR method, respectively (table 4). These results suggest that the district target populations may be too small or that over-reporting of vaccinations may occur. Similarly, a high proportion of subnational units have unlikely low coverage rates, even though DPT1 coverage rates are expected to be high almost everywhere according to survey data. Possible explanations are overestimation of target populations, under-reporting of events or numerator/denominator mismatches.

**Table 4:** Percentage of districts with coverage over 100% and of districts with coverage at least 15% lower than national level, using official projections of population and births by district, 2017

Country	ANC1 coverage >100% based on		ANC1 coverage at least 15% lower based on		DPT1 coverage >100% based on		DPT1 coverage at least 15% lower based on	
									Births	CBR	Births	CBR	Births	CBR	Births	CBR
Botswana	–	–	–	–	–	–	–	–
Burundi	83	70	15	39	41	43	15	22
Eritrea	95	12	28	33	95	9	25	24
Kenya	15	34	19	21	17	43	11	17
Lesotho	20	40	30	20	0	0	10	20
Malawi	0	0	0	0	7	45	28	28
Mozambique	7	91	21	51	2	63	0	35
Namibia	–	21	–	47	–	59	–	47
Rwanda	80	23	13	23	93	37	7	20
South Sudan	100	–	50	–	100	–	50	–
Tanzania	55	71	58	32	53	73	15	21
Uganda	15	66	19	25	25	79	19	28
Zambia	30	76	23	28	30	73	29	33
Zimbabwe	6	44	16	10	37	48	40	41
Median	25	42	20	27	33	46	17	26
IQR	68	47	13	13	48	25	18	13

ANC, antenatal care; CBR, crude birth rate; DPT, diphtheria-pertussis-tetanus.

The choice of the denominator is based on multiple arguments. If the differences between service-based and census-based estimates of target populations are small, it is best to use the census-based projections, particularly for national and region/provincial level. However, national consistency does not necessarily mean that these denominators work well for all districts. Ultimately, the choice needs to be made based on an individual district analysis that may lead to the identification of groups of districts for which the population projections do not perform as well as target populations. Kenya and Rwanda provided examples of the use of facility reports (DPT1 and BCG, respectively) in endline analyses to improve the estimation of target populations and coverage rates.

## Analysis

A clean health facility data set should form the basis for analyses that are presented in annual reports and other formats to inform monitoring of progress and annual reviews, and evidence-based policy and programme planning. Several countries rank districts according to coverage rates or indexes of performance (eg, Uganda). Further analyses may include quantifying district-level estimates of populations reached and not reached with specific interventions and comparisons of district health outputs with health system and other inputs.^11 27 28^ In addition, the combination of analyses and presentation of statistics from survey and facility reports enables a more complete interpretation of facility data-based statistics, but was not done on a regular basis in any of the 14 countries.

In future, analyses using geospatial or other advanced methods could help generate predicted values that could serve as a method to assess the plausibility and quality of statistics that are generated from health facility data, especially at the district level.^11 13 29^

## Conclusion

The assessment of health facility data from 14 countries of the Eastern and Southern Africa region showed the potential of such data for regular (sub)national health statistics. The introduction of web-based digital platforms that facilitate the analysis, use and visualisation of health facility data at the district level appears to lead to gradual improvements in data quality, especially completeness of reporting, and enables a systematic approach of data quality assessment and analysis. Yet, major gaps remain. First, as shown with the data from the 14 countries, there are major data quality problems that need to be addressed in the coming years, including improvement of estimation of target populations. Several studies have described the problems and implemented ways to improve the quality of routine data with varying success, including training of health workers, strengthening of feedback, introduction of case-based electronic management systems, data verification surveys and other interventions.^5 14 30–32^

Second, in most countries, use of facility data is restricted to a limited number of individuals. Five countries indicated that they provide a wider public access based on an access password on request. The access to health data facility, information distribution and promotion of culture of information are critical for improving health information systems and health status more broadly. Facility data are promising sources of statistics for evidence-based decision making, planning and advocacy.^33 34^ Less restrictive and systematic access to data also stands for transparency about data processing and quality.

Third, data quality assessment and computation of credible statistics from health facility data are not straightforward. Technology has advanced much faster than data quality improvements. Currently, country capacities to deal with health facility data, carry out data quality assessment and adjustments and produce credible statistics are still limited. National analysts in the Ministry of Health, public health institutions and national statistical offices need to have access to an optimal set of tools and skills to analyse and synthesise health facility data and produce the best possible statistics with well-documented audit trails.

The use of data from RHIS, to improve health system performance or to make evidence-based decisions, remains suboptimal in many developing countries in Africa and Asia.^35^ The Performance of Routine Information System Management (PRISM) framework describes the factors linked to access, quality and use of data and the lack of ‘information culture’ in those countries.^33 35^ RHIS is defined as a complex system in the PRISM framework, and its improvement requires to bring together and take into account the role and relationships between the technical, organisational or environmental and behavioural factors to improve routine health data quality and use of health information in order to strengthen the health system and population health status as an ultimate goal.^33^ There are improvements in the data culture as evidenced by countries’ interest in scorecards and the use of WHO data quality module incorporated in DHIS2.

The technological advances provide a major opportunity to further strengthen data quality and analyses of health facility data at local and national levels in the coming years. Improved statistics from health facility data are a critical step towards evidence-based planning and targeting of programme on the road to universal health coverage of essential interventions.
