# Supplementary material for: Generating statistics from health facility data: the state of routine health information systems in Eastern and Southern Africa
Source: BMJ Glob Health. 2019 Sep 29;4(5):e001849. doi: 10.1136/bmjgh-2019-001849 (PMC6768347; doi:10.1136/bmjgh-2019-001849)
Supplement: Supplementary data [file bmjgh-2019-001849supp001.pdf]

Annex 1: Ratio between reported number of ANC1 and reported number of DPT1 over time and by country (%)

Country	2013	2014	2015	2016	2017
Botswana	36	161	775	1,338	2,557
Burundi	145	143	141	125	125
Eritrea	107	101	108	98	113
Kenya	98	100	101	102	106
Lesotho	-	98	101	101	99
Malawi	158	60	40	37	30
Mozambique	-	-	-	197	286
Namibia	-	-	118	108	106
Rwanda	113	109	110	102	101
South Sudan	-	264	115	110	99
Tanzania *	123	112	110	110	114
Uganda	-	-	99	100	103
Zambia	92	106	103	105	105
Zimbabwe	102	103	106	104	106
**Median**	**107**	**106**	**108**	**104**	**106**

** 2014 to 2018, reference years for Tanzania*
